# Prolonged persistence of IgM against dengue virus detected by commonly used commercial assays

**DOI:** 10.1186/s12879-018-3058-0

**Published:** 2018-04-02

**Authors:** Yu-Wen Chien, Zi-Hu Liu, Fan-Chen Tseng, Tzu-Chuan Ho, How-Ran Guo, Nai-Ying Ko, Wen-Chien Ko, Guey Chuen Perng

**Affiliations:** 1 Department of Public Health, College of Medicine, National Cheng Kung University, Tainan, Taiwan; 2 Department of Occupational and Environmental Medicine, National Cheng Kung University Hospital, College of Medicine, National Cheng Kung University, Tainan, Taiwan; 3 Department of Environmental and Occupational Health, College of Medicine, National Cheng Kung University, Tainan, Taiwan; 4 Department of Nursing, National Taipei University of Nursing and Health Sciences, Taipei, Taiwan; 5 National Institute of Infectious Diseases and Vaccinology, National Health Research Institutes, Tainan, Taiwan; 6 Institute of Basic Medical Sciences, College of Medicine, National Cheng Kung University, Tainan, Taiwan; 7 Department of Nursing, College of Medicine, National Cheng Kung University, Tainan, Taiwan; 8 Department of Medicine, College of Medicine, National Cheng Kung University, Tainan, Taiwan; 9 Department of Microbiology and Immunology, College of Medicine, National Cheng Kung University, Tainan, Taiwan

**Keywords:** Flavivirus, Immunoglobulin M, Dengue, Diagnosis

## Abstract

**Background:**

Initial symptoms of dengue fever are non-specific, and thus definite diagnosis requires laboratory confirmation. Detection of IgM against dengue virus (DENV) has become widely used for dengue diagnosis. Understanding the persistence of anti-DENV IgM in subjects after acute infection is essential in order to interpret test results correctly. Although the longevity of anti-DENV IgM has been vehemently investigated in symptomatic children, anti-DENV IgM persistence in adults and in asymptomatically infected people have seldom been reported.

**Methods:**

We prospectively investigated 44 adults with detectable anti-DENV IgM in a serosurvey conducted in the 2015 dengue epidemic in Tainan, Taiwan. Among subjects within the cohort, 17 were classified to be symptomatic and 27 were asymptomatic. The enzyme-linked immunosorbent assay (ELISA) from Standard Diagnostic (SD) and Focus Diagnostic were used to detect anti-DENV IgM for specimens collected initially, at 6 and 12 months. Regression analyses were used to estimate the duration of anti-DENV IgM fell below the detectable level. Rapid dengue tests from Standard Diagnostics had been widely adopted to detect anti-DENV IgM in Taiwan during the 2015 dengue outbreak. As such, collected specimens were also evaluated with the SD rapid dengue test in parallel.

**Results:**

Anti-DENV IgM was detectable in 70.5 and 46.2% of the 44 subjects at 6 months and 12 months by the SD ELISA, respectively, while 13.6 and 7.7%, respectively, by the Focus ELISA. There was no significant difference in anti-DENV IgM detection for the follow-up specimens between subjects with symptomatic and asymptomatic infections. The regression analysis estimated that anti-DENV IgM persistence fell to the undetectable level at 338.3 days (95% CI 279.7–446.9) by SD ELISA, while at 175.7 days (95% CI 121.9–221.1) by Focus ELISA. The detectable frequency of anti-DENV IgM by rapid tests was 86.4%, 68.2 and 35.9% at initial, 6 and 12 months, respectively.

**Conclusion:**

Anti-DENV IgM was found to persist much longer than previously thought, suggesting a necessity of re-evaluation of the use of anti-DENV IgM for both the diagnosis of dengue and serological surveillance, especially when large outbreaks have occurred in the preceding year.

## Background

Dengue, one of the most common arbovirus infections in human, is caused by the infections of four dengue virus (DENV1–4) serotypes [1]. Dengue has become a major public health threat because of greatly increased disease incidence and geographical expansion in recent decades [1]. Currently, approximately 3.9 billion people living in 128 countries are at risk of DENV infection [2]. A recent estimate suggested that DENV resulted in 58.4 million symptomatic cases annually and was responsible for 1.14 million disability-adjusted life-years in 2013 [3].

Initial symptoms of dengue are non-specific, and thus definite diagnosis requires laboratory confirmation. Accurate, inexpensive, and timely diagnostic tools by using a single specimen are essential for patient care, surveillance, outbreak investigation and control [4, 5]. Dengue viremia can be detected during the early febrile period, usually from 0 to 7 days following symptom onset by virus isolation, viral nucleic acid or antigen detection [5]. However, since many affected subjects seek for healthcare quite late, the programmatic detection of viral materials is a challenging task. Therefore, alternative diagnosis of dengue by detection of anti-DENV immunoglobulin (Ig) M using capture enzyme-linked immunosorbent assays (MAC-ELISA) has become widely used due to the timing of IgM appearance. In addition, this assay is relatively inexpensive and less labor intensive, as compared to viral isolation or nucleic acid detection [4, 6]. Anti-DENV IgM can be detected as early as 3–5 days and reaches its peak around 12–14 days after symptom onset [6, 7]. Detection of IgM is a preferred diagnostic test when the specimen is collected 5 days after disease onset [4, 6].

Delineating the duration for the detection of anti-DENV IgM after infection is essential for diagnosis and research. Studies in the 1980s and 1990s using MAC-ELISA showed that anti-DENV IgM persisted for about 2–3 months after symptom onset [8–10]; another study in 1997 collected blood samples from children 6 months after acute DENV infection and found anti-DENV IgM antibody fell to undetectable level [11]. Therefore, numerous reviews and guidelines stated that anti-DENV IgM only persisted for 2–3 months [4, 5, 12–14]. However, the diagnostic tools used in the last century might be less sensitive than what are used today. More recently, Prince et al. from Focus Diagnostics (Cypress, CA, USA) used their MAC-ELISA and estimated that anti-DENV IgM antibodies persisted longer than that which was initially reported in the 1980s and 1990s by regression analysis - approximately 179 days for patients with primary infection and 139 days for those with secondary infection [15].

Rapid diagnostic tests (RDTs) for dengue based on immunochromatographic methods to detect NS1 antigen with or without anti-DENV IgM and IgG antibodies have become increasing convenient and available. They are inexpensive, easy to perform without the need for additional laboratory facilities, and can provide results within 15 min. The sensitivity of the NS1 RDTs ranges from 38 to 71% [16]; combining the NS1 and IgM results may improve the overall sensitivity from 49 to 93% [17].

Most of DENV infection was asymptomatic or subclinical [1]. To our knowledge, however, whether the persistence of anti-DENV IgM differs between people with asymptomatic and symptomatic infection has seldom been explored. In addition, previous studies investigated anti-DENV IgM persistence mostly in symptomatic children [8, 11] or did not specify the age distribution [9, 10]. The anti-DENV IgM persistence in adults is less well known. Furthermore, the readouts obtained from the measurement of anti-DENV IgM in hyper-endemic countries may have difficulty in differentiation between the left over from a previous infection and a recent re-infection during the follow-up period. Tainan is a city with a population of approximately 1.88 million located in southern Taiwan, where dengue is not considered to be endemic. A severe dengue epidemic caused by DENV2 occurred in 2015, resulting in more than 22,000 confirmed cases, mostly adults [18, 19]. A seroprevalence survey was conducted in the general public during the declining phase of this epidemic in 2015. The aim of this study was to investigate the persistence of anti-DENV IgM antibodies among adults who were tested IgM-positive in this serosurvey and to examine whether the anti-DENV IgM persistence differed between symptomatically and asymptomatically infected adults by using two commercial ELISA tests and one RDT.

## Methods

### Participants

The 2015 dengue epidemic in Tainan began in May, peaked in September, and rapidly declined in October. A seroprevalence survey was conducted to recruit healthy volunteers in selected districts and in a university between mid-October and November. The participants had to fill in a short questionnaire which inquired whether they had been diagnosed with dengue in 2015; if the answer was yes, the month of month of disease onset was recorded. In Taiwan, reporting of people with suspected dengue virus infection is required by law. During this epidemic, free NS1 rapid tests with and without combining IgM/IgG had been widely adopted in hospitals and clinics and those with symptoms suspicious of dengue but negative NS1 results were advised to receive further laboratory tests to confirm. Therefore, the great majority of people who reported being diagnosed with dengue in 2015 should have received laboratory confirmation, not just clinical diagnosis. Those who were tested IgM-positive in the serosurvey were contacted by phone approximately 6 months and 12 months after the first blood drawing; blood samples were taken after signing the informed consent.

### Diagnostic assays

Dengue IgM capture ELISAs manufactured by Standard Diagnostics (SD; Kyonggi-do, South Korea) were used in the initial serosurvey to identify anti-DENV IgM-positive subjects; samples collected at the 6-month and 12-month follow-up were also tested using this commercial kit. To compare our results with the study conducted by Prince et al. [15], Dengue Virus IgM Capture DxSelect manufactured by Focus Diagnostics was also used. Samples were tested in duplicate and interpreted according to the manufacturers’ instruction. Furthermore, the cut-off values in current study were implemented according to the instructions accompanied with the assays. The cut-off value for the SD ELISA was the mean optical density (OD) at 450 nm of the negative controls plus 0.30; any sample with a mean OD no less than the cut-off value was considered positive. To calculate the sample index values for the Focus ELISA, specimen mean OD values corrected for the blank readings were divided by the mean of cut-off calibrator absorbance values. An index value of greater than 1.0 was considered positive.

SD BIOLINE Dengue Duo rapid dengue tests had been widely adopted to detect anti-DENV IgM globally and more recently in Taiwan during the 2015 dengue outbreak. As such, collected specimens were also evaluated with the SD rapid dengue test in parallel. In order to interpret the readout results, three classes were defined: definitely positive, very faint, and negative. All the tests were performed with serum samples.

### Statistical analysis

The percentages of detectable anti-DENV IgM in collected specimens at different time points for the two ELISA kits were calculated and tabulated according to age, self-report dengue diagnosis history in 2015, and initial anti-DENV IgM level, which was defined by subtracting the mean OD values of the negative controls in each plate from the mean sample OD values using the SD ELISA (higher or lower than median). Categorical variables were compared using Fisher’s exact tests. For those who reported being diagnosed with DENV infection in 2015, it was assumed that the middle dates of the reported months of disease diagnosis were their symptom onset dates. Linear regression was used to model the log-transformed mean OD values (for the SD ELISA) or index values (for the Focus ELISA) as a function of the number of days after disease onset to estimate when anti-DENV IgM fell below the undetectable level. Analyses were performed using SAS 9.4 (SAS Institute, Cary NC). Results were considered statistically significant at the *p* < 0.05 level.

## Results

A total of 1520 healthy volunteers were recruited in the serosurvey conducted in late 2015; ninety-eight subjects were defined as anti-DENV IgM-positive by the SD ELISA. Since only 44 of these subjects agreed to participate at the 6-month follow-up, we therefore focused on them in this study. There was no significant difference in age (*p* = 0.1276) between those who agreed to participate in the follow-up study and those who refused. However, females appeared to be much more dominant in the follow-up study (*p* = 0.0043). The average age of the 44 participates was 55.0 years (range 23–74 years); 37 (84.1%) were female (Table 1). Five subjects dropped out of the study at the 12-month follow-up (Table 1).

**Table 1:** Detection of anti-DENV IgM by two commercial ELISA tests at initial survey, 6 and 12 months

	No. DENV IgM positive/total (%)^a^					
	Initial		6 months later		12 months later	
	SD^b^	Focus^c^	SD^b^	Focus^c^	SD^b^	Focus^c^
All	44/44 (100.0)	40/44 (90.9)	31/44 (70.5)	6/44 (13.6)	18/39 (46.2)	3/39 (7.7)
p-value^d^			< 0.0001		< 0.0001	
Age						
20–50	12/12 (100.0)	8/12 (66.7)	9/12 (75.0)	2/12 (16.7)	5/10 (50.0)	1/10 (10.0)
51–60	16/16 (100.0)	16/16 (100.0)	12/16 (75.0)	2/16 (12.5)	7/16 (43.8)	1/16 (6.3)
> 60	16/16 (100.0)	16/16 (100.0)	10/16 (62.5)	2/16 (12.5)	6/13 (46.2)	1/13 (7.7)
p-value^d^	–	–	0.7676	> 0.9999	> 0.9999	> 0.9999
Sex						
Male	7/7 (100.0)	7/7 (100.0)	6/7 (85.7)	1/7 (14.3)	2/5 (40.0)	1/5 (20.0)
Female	37/37 (100.0)	33/37 (89.2)	25/37 (67.6)	5/37 (13.5)	16/34 (47.1)	2/34 (5.9)
p-value^d^	–	–	0.6542	> 0.9999	> 0.9999	0.3452
Initial IgM level^e^						
> Median	22/22 (100.0)	22/22 (100.0)	19/22 (86.4)	5/22 (22.7)	12/19 (63.2)	3/19 (15.8)
< Median	22/22 (100.0)	18/22 (81.8)	12/22 (54.5)	1/22 (4.5)	6/20 (30.0)	0/20 (0.0)
p-value§	–	–	0.0452	0.1853	0.0562	0.1060
Diagnosed cases						
Yes	17/17 (100.0)	16/17 (94.1)	11/17 (64.7)	1/17 (5.9)	7/15 (46.7)	1/15 (6.7)
No	27/27 (100.0)	24/27 (88.9)	20/27 (74.1)	5/27 (18.5)	11/24 (45.8)	2/24 (8.3)
p-value^d^	–	–	0.5205	0.3801	> 0.9999	> 0.9999

^a^Number of positive anti-DENV IgM/number of specimens tested (percentage)

^b^ELISA kits manufactured by Standard Diagnostics

^c^ELISA kits manufactured by Focus Diagnostics

^d^The difference in the detection rate among different groups were examined using Fisher’s exact tests

^e^The initial anti-DENV IgM level was defined by subtracting the mean the OD values of the negative controls in each plate from the mean OD values of the samples using the SD ELISA

Discordant results were observed between the two commercial anti-DENV IgM ELISA kits. For the SD ELISA, 70.5 and 46.2% of the 44 subjects remained anti-DENV IgM-positive at 6 and 12 months, respectively (Table 1). As for the Focus ELISA, 90.9% of the 44 initial samples were anti-DENV IgM-positive; in follow-up samples, the percentages of detectable anti-DENV IgM antibodies at 6 and 12 months were 13.6 and 7.7%, respectively (Table 1). The differences of IgM detection between SD and Focus ELISA kits were statistically significant for samples collected at the two follow-ups (both *p* < 0.0001) (Table 1). Detection of anti-DENV IgM did not vary by age and sex at 6 months and 12 months no matter which ELISAs were used (Table 1). Subjects with higher initial level of anti-DENV IgM defined by the SD ELISA were significantly more likely to have detectable anti-DENV IgM at 6 months by the same kit (86.4 and 54.5%, *p* = 0.0452), but the detection difference became non-significant at 12 months (63.2 and 30.0%, *p* = 0.0562). In addition, the initial samples with anti-DENV IgM level higher than the median defined by the SD ELISA were all detected by the Focus ELISA; in contrast, 4 of the specimens with IgM level lower than the median were not detected by the Focus ELISA (Table 1).

Seventeen (38.6%) of the 44 subjects reported being diagnosed with dengue in 2015. Based on the subject’s recall, the median duration from the estimated symptom onset dates to the blood samples drawn was 51 days (range 9–98 days). There was no significant difference in the persistence of anti-DENV IgM at 6 months and 12 months between people with and without dengue diagnosis in 2015 for both ELISA tests (Table 1).

**Fig. 1:**
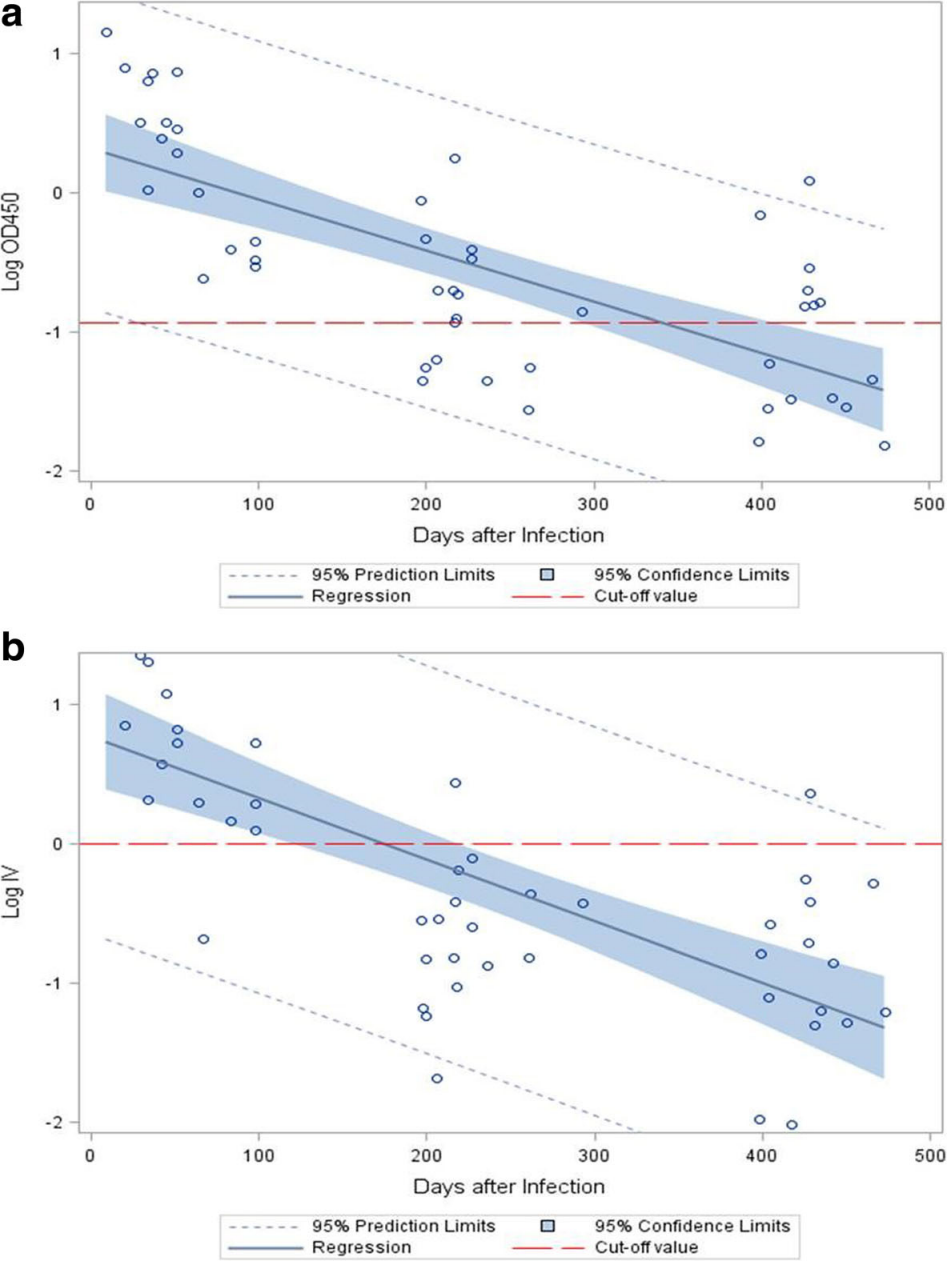
Kinetic scatterplots and regression analyses of log-transformed mean optical density values at 450 nm (OD450) and mean index values (IV) for anti-DENV IgM measured by the SD ELISA and the Focus ELISA, respectively, over time in 17 people who were anti-DENV IgM positive initially defined by the SD ELISA and reported being diagnosed with DENV infection in 2015. **a** Results of the SD ELISA. The dashed line represents the mean cut-off OD value among different plates. (R-square: 0.5176; slope coefficient: − 0.0037, *p* < 0.0001). **b** Result of the Focus ELISA. The dashed line represents the cut-off index value. (R-square: 0.5057; slope coefficient: − 0.0044, p < 0.0001)

The decay kinetics of the anti-DENV IgM in the 17 subjects who reported being diagnosed with dengue were delineated by scatter plots with the results from both ELISA kits (Fig. 1). Regression analyses showed that the logarithm of the mean OD values for the SD ELISA (Fig. 1a) and the mean index values for the Focus ELISA (Fig. 1b) decayed at rates of 0.0037 (95% CI 0.0026–0.0047) per day and 0.0044 (95% CI 0.0031–0.0057) per day, respectively. It was estimated that anti-DENV IgM became undetectable after 338.3 days (95% CI 279.7–446.9) by the SD ELISA and 175.7 days (95% CI 121.9–221.1) by the Focus ELISA.

Results from the SD RDTs were interpreted as definitely positive in 63.6%, 18.2 and 5.1% initially, at 6 months and 12 months, respectively (Table 2). If very faint bands on RDTs were also interpreted as positive according to manufacturer’s instruction, anti-DENV IgM were detectable in 86.4%, 68.2 and 35.9% initial, at 6 months, and 12 months (Table 2).

**Table 2:** Detection of anti-DENV IgM by rapid tests from Standard Diagnostics at initial survey, 6 and 12 months

	No. DENV IgM positive/total (%)^a^		
	Initial	6 months	12 months
Definitely positive	28/44 (63.6)	8/44 (18.2)	2/39 (5.1)
Very faint	10/44 (22.7)	22/44 (50.0)	12/39 (30.8)
Negative	6/44 (13.6)	14/44 (31.8)	25/39 (64.1)

^a^Number of positive anti-DENV IgM/number of specimens tested (percentage)

## Discussion

This study investigated the persistence of anti-DENV IgM among adults who were infected in a severe dengue epidemic in Tainan in 2015. The main circulating serotype was DENV2 and approximately 90% of the acute patients suffered from primary DENV infection [19]. The results demonstrated that there was a discordance in the length of anti-DENV IgM detection by commonly used commercial ELISA tests. The estimated duration that anti-DENV IgM became undetectable by the Focus ELISA was 175.7 days after symptom onset in adults, which was very similar to the results reported by Prince et al., though the age distribution in their study was unknown [15]. In contrast, regression analysis showed that the anti-DENV IgM persisted for almost 1 year by the SD ELISA, and nearly half of the participants had detectable anti-DENV IgM more than 1 year post infection. Although the discordant outcomes were a surprised finding, at this moment, we don’t know the reasons contributing to the difference in readouts. Although detection of anti-DENV IgM in one single specimen does not provide a definite diagnosis of dengue, it has been widely used, especially when blood samples are taken more than 5 days after disease onset and in non-endemic countries [4, 6]. Furthermore, it is usually difficult to obtain second convalescent specimens. However, if there is a large dengue epidemic in the previous year, the recognition of anti-DENV IgM in symptomatic patients without virology confirmation may represent anti-DENV IgM persistence from the previous year rather than an acute infection; thus alternative etiologies should be pursued and clinical management consequently be altered. Also, the interpretation of faint anti-DENV IgM bands on RDTs after a large dengue outbreak in the preceding year should be more conservative. In addition to patient care, serological surveillance by testing of those who link to the confirmed cases epidemiologically to search for additional people with both symptomatic and asymptomatic DENV infection has also been advocated in non-endemic areas to break the transmission cycles and contain epidemics in the early stages [20]. Nevertheless, the value of anti-DENV IgM for outbreak investigation and surveillance may also be complicated or even hindered if a large epidemic has occurred in the preceding year. Our study also provides important new information to the field of dengue research, especially for scholars who commonly use these tests to interpret and address their study findings.

Previous studies barely examined anti-DENV IgM persistence among people asymptomatically infected with DENV because of the difficulty in knowing when the initial infection occurred. In our study, we found that the anti-DENV IgM persistence did not differ between those who reported being diagnosed with DENV infection in 2015 and those who did not. In Taiwan, notification of patients with suspected DENV infection by physicians is mandatory; one study which investigated the quality of the notification system found that the overall reporting rate was 86.6% during 2006–2007 [20, 21]. In 2015, there was an extraordinarily high awareness of dengue and febrile illness among healthcare professionals and general public due to pervasive dengue educational campaigns during this unprecedented dengue epidemic in Tainan; RDTs were also widely used in patients with any symptoms suspicious of DENV infection [22]. Therefore, the under-reporting of the symptomatic cases has been very low in Taiwan and probably was even lower in 2015. Thus, the majority of anti-DENV IgM positive volunteers without dengue diagnosis in our study could be characterized as asymptomatic infections. Our study thus provides evidence that the persistence of anti-DENV IgM does not differ between asymptomatic and asymptomatic infections.

In our study, it seems that the SD ELISA has higher anti-DENV IgM positive rate than the Focus ELISA. The performance of the two ELISAs has been evaluated previously [23, 24]. One study demonstrated that the two tests were similar in sensitivity [23], while Domingo et al. showed that the Focus IgM ELISA seemed to have higher sensitivities than the SD ELISA [24]. However in that study, the tests were performed by different laboratories, some of which might be less experienced in dengue diagnosis and thus their results might not be directly comparable [24]. Additionally, Domingo et al. examined samples from patients infected with DENV1 and DENV3 [24] while the main circulating serotype in our study was DENV2 [19], which may also partially explain the difference. We used SD ELISA in our original serosurvey to define IgM positivity and the aim of this follow-up study was to investigate IgM persistence, not to evaluate the sensitivity of the two tests. To compare their sensitivity directly, all the anti-DENV IgM-negative samples defined by the SD ELISA in the original serosurvey should be tested by Focus ELISA simultaneously. As for the specificity, the SD ELISA has very high specificity and shows no cross-reactivity with other similar flaviviruses including Japanese encephalitis virus and West Nile virus [23]. Although false positive results may be present in people with malaria or previous DENV infection [23], Taiwan was certified by the World Health Organization (WHO) as “malaria-free” in 1965 [25] and the majority of DENV-infected people in this epidemic suffered from primary infection [19]. Furthermore, although the current ELISA kits may have a concern of cross-reactivity to Zika virus infection, there are no local Zika cases reported in Taiwan as of today. Consequently, the false positive rate in our study should be low.

The major strength of this study is that the samples were collected in a non-endemic area and there was no dengue outbreak in the following year, 2016, despite extensive surveillance efforts. We therefore can be sure that detection of anti-DENV IgM in the follow-up samples was a result of IgM persistence rather than re-infection of DENV during follow-up. However, there were also several limitations in our study. Firstly, we assumed that the middle date of the self-reported month of diagnosis was the symptom onset for each subject. Although the assumed onset dates were based on individual’s recall and thus subject to error, this should not bias our results given the time span considered in the regression analysis. Secondly, although there might be misclassification using self-reporting of history of dengue diagnosis as a surrogate to define symptomatic and asymptomatic infection, the magnitude of the bias should be low as previously mentioned. Thirdly, most of the study subjects were adults suffered from primary DENV2 infection, so our results may not be generalized to children living in endemic regions and/or other serotypes. Further studies which include a wider age span, more dengue serotypes, and secondary infection are recommended. Fourthly, the sample size in this study was small, especially for the regression analysis. Finally, although the current two ELISA assays are widely utilized for acute dengue diagnosis, we do not know which assay is better for persistent anti-DENV IgM measurement, since currently there is no gold standard test for persistent anti-DENV IgM. As such, despite our findings presume to be very important for dengue diagnosis, we are still unable to affirm whether a person is anti-DENV IgM positive or not if his or her anti-DENV IgM is detectable by SD ELISA kit but undetectable by Focus ELISA kit. Subsequently, the biology of immune response after certain period of dengue virus infection could be far more complex and warrants further investigation.

## Conclusions

It was found that anti-DENV IgM detected by some current commercial ELISA kits or RDTs persisted much longer than previously thought, which may complicate diagnosis of dengue and surveillance efforts in the following year after a large dengue epidemic. Therefore, diagnosis of dengue using anti-DENV IgM following large dengue outbreaks should be more conservative. Our study also demonstrated that the duration of anti-DENV IgM persistence did not differ between symptomatically and asymptomatically infected individuals, which has seldom been reported in the literature.

## Abbreviations

DENV: Dengue virus
ELISA: Enzyme-linked immunosorbent assay
RDT: Rapid diagnostic test
